# Interplay Between NLRP3 Activation by DENV-2 and Autophagy and Its Impact on Lipid Metabolism in HMEC-1 Cells

**DOI:** 10.3390/pathogens14121292

**Published:** 2025-12-16

**Authors:** Giovani Visoso-Carvajal, Julio García-Cordero, Yandy Ybalmea-Gómez, Margarita Diaz-Flores, Moisés León-Juárez, Rosaura Hernández-Rivas, Porfirio Nava, Nicolás Villegas-Sepúlveda, Leticia Cedillo-Barrón

**Affiliations:** 1 Departamento de Biomedicina Molecular, Centro de Investigación y Estudios Avanzados (Cinvestav-IPN) Av. IPN # 2508 Col. San Pedro Zacatenco, Ciudad de México 07360, Mexico; carvajalgv@gmail.com (G.V.-C.); jugarcia@cinvestav.mx (J.G.-C.); yandy.ybalmea@cinvestav.mx (Y.Y.-G.); rohernan@cinvestav.mx (R.H.-R.); nvillega@cinvestav.mx (N.V.-S.); 2 Unidad de Investigación Médica en Bioquímica, Instituto Mexicano del Seguro Social, Ciudad de México 06600, Mexico; mardiaz2001@yahoo.com; 3 Laboratorio de Virologia Perinatal, Departamento de Inmunobioquimica, Instituto Nacional de Perinatologia, Ciudad de México 11000, Mexico; moisesleoninper@gmail.com; 4 Departamento de Fisiología, Biofísica y Neurociencias, Cinvestav-IPN, Ciudad de México 07360, Mexico; pnava@cinvestav.mx

**Keywords:** NLRP3, SREBP-1, SREBP-2, fatty acid synthase, Beclin-1, LC3, glyburide

## Abstract

Dengue Virus (DENV) induces assembly of the NOD-like receptor (NLR) family pyrin domain containing-3 (NLRP3) inflammasome and autophagy, which are closely interconnected processes playing crucial roles in lipid metabolism and DENV replication. However, the autophagy–NLRP3 activation interplay during DENV infection in human endothelial cells remains incompletely understood. We aimed to elucidate effects of NLRP3 activation on autophagy during DENV-2 infection. We investigated how autophagy-related molecules are altered by NLRP3 inhibition and how this regulation affects lipid metabolism, through the master lipid transcription factors SREBP-1 and 2, which increase the expression of their target lipid-synthesizing genes such as fatty acid synthase (FAS) in a model of microvascular endothelial cells (HMEC-1). We demonstrated a dynamic interplay between inflammasome activity and autophagy in DENV-infected HMEC-1 cells: autophagy increases early during infection and decreases as inflammasome activity increases. NLRP3 inflammasome inhibition affects viral replication. Glyburide (an inflammasome inhibitor) treatment partially inhibited DENV-induced NLRP3 inflammasome activation. Non-structural viral protein expression (NS3 and NS5) and infectious viral-particle formation were significantly reduced. NLRP3 inhibition also downregulated SREBP-1 and SREBP-2 activation. These findings provide new insights into the modulation of the interconnected NLRP3 inflammasome, autophagy, and lipid metabolism pathways, presenting a promising therapeutic strategy for severe clinical forms of dengue.

## 1. Introduction

Dengue virus (DENV), a member of the Flaviviridae family, is an RNA virus transmitted by infected *Aedes* mosquitoes. This virus causes dengue, which is the more common arboviral disease, with approximately 100–400 million people infected each year worldwide. There has been limited progress in vaccine development, and thus far, no specific drug for dengue has been reported. Thus, it is necessary to understand the involvement of the virus in dengue pathogenesis. DENV is known to manipulate the cellular metabolism of target cells to support their replication. These viruses regulate processes including the cell cycle, vesicular trafficking, lipid metabolism, and the activity of associated enzymes [1,2,3,4]. Viruses also modulate the expression of protein complexes, such as the inflammasome and autophagy-related proteins, which they exploit to promote their replication and evade the immune system [5,6].

The activation of the NOD-like receptor (NLR) family pyrin domain containing-3 (NLRP3) inflammasome is important in severe DENV infection. NLRP3 functions as a molecular platform within innate immunity and is activated by viruses, such as DEN and Zika virus (ZIKV) [7,8]. The NLRP3 inflammasome comprises an NOD receptor, its adapter protein (ASC), and procaspase-1 [9,10]. NLRP3 activation requires two signals: the first is mediated by Toll-like receptor (TLR) and RIG-I-like receptor (RLR) signaling, while the second promotes procaspase-1 recruitment to NLRP3 and its subsequent maturation. A study reporting NLRP3 activation by DENV-2 [10] observed that infected macrophages exhibited upregulated NLRP3 transcription and increased caspase-1 activity. In addition to a proinflammatory response, which contributes to disease immunopathogenesis, there are evidences that indicates the role of inflammasome in the regulation of other virus-induced cellular processes such as pyroptosis and recruitment of immune cells [11]. Autophagy is a multifunctional cellular process involved in degradation and recycling of cellular components, in response to different types of stress [12], and it occurs in three main steps: (i) nucleation, (ii) elongation of vesicles to form the phagophore, which later (iii) matures into the autophagosome. Autophagy impairment leads to the excessive or reduced activation of the NLRP3 inflammasome. Thus, inflammasome and autophagy are closely regulated to maintain cellular homeostasis.

Multiple protein complexes regulate autophagy, including Unc-51, ULK1, PI3K, PIK3C3/Vps34 and Beclin1, which are essential for producing phosphatidylinositol 3-phosphate, as well as the ubiquitin-like conjugation system, which includes autophagy-related gene 5 (ATG5) and the microtubule-associated protein 1 light chain 3 (LC3). LC3 is a key autophagy protein; once it suffers lipidation, it gives rise to LC3-II, which is a stable marker for monitoring autophagy. Furthermore, DENV induces lipophagy in THP1 cells and monocytes; it activates heat shock factor, which in turn upregulates ATG7 and enhances autophagic activity [13]. Autophagy also contributes to the innate immune response by degrading misfolded proteins and damaged organelles.

Furthermore, cumulative evidence indicates that both inflammasome activation and autophagy are tightly linked to lipid metabolism, which is essential for efficient DENV replication [14,15]. Lipids are essential molecules for both the host and virus, serving as energy sources and structural components. Sterol Regulatory Element Binding Proteins (SREBPs) are precursor proteins in the endoplasmic reticulum (ER) membrane. Upon cleavage, a transcriptional fragment translocates to the nucleus, to regulate the biosynthesis of cholesterol, triglycerides, phospholipids, and fatty acids via the MAPK pathway. To date, three SREBP proteins have been described: SREBP-2, SREBP-1a, and SREBP-1c [16]. The last two are potent activators of genes involved in fatty acid and cholesterol synthesis [17,18,19,20]. In contrast, SREBP-2 mediates only cholesterol biosynthesis [17,18]. Viruses can manipulate the expression of molecules involved in lipid metabolism, such as fatty acid synthase (FAS) or 3-hydroxy-3-methylglutaryl-CoA reductase (HMGCR), to favor viral replication [18,20].

Notably, SREBPs and inflammasome activation are prominent phenomena in viral diseases, like COVID-19. SARS-CoV-2 has been reported to upregulate both SREBP and inflammasome activation, which support viral replication [15,21,22]. Additionally, Zika virus increases SREBP-2 expression with proviral effects [16,23]. SREBP-1a has been shown to activate NLRP gene transcription in macrophages, linking lipogenesis with the innate immune response during endotoxic shock [24]. Furthermore, rotavirus A (RVA) infection can reprogram SREBP-dependent lipogenic pathways in virus-infected cells; silencing SREBP significantly reduces viral protein synthesis, genome replication, and progeny virus production [25].

The specific role of NLRP3 inflammasome activation in regulating autophagy and lipid metabolism during DENV infection of endothelial cells (which are key cells involved in the pathogenesis of severe DENV infections) has been poorly studied. Thus, in the present study, we evaluated effects of DENV-activated NLRP3 on autophagy components and viral replication. Both processes are closely linked to lipid metabolism through SREBP transcription factors. Thus, we inhibited NLRP3 activation by DENV through glyburide treatment of DENV-infected endothelial cells of microvasculature (HMEC-1) and evaluated the effect on autophagy and lipid transcription factors. Our results suggest that DENV infection modulates autophagy at an early stage, and NLRP3 is activated at a later stage along with the activation of master genes in lipogenesis (SREBP1 and 2) to generate sufficient ATP for viral replication

This approach allowed us to gain a better understanding of the mechanisms of NLRP3 in DENV infection and its influence on lipid metabolism in HMEC-1 cells. The targeting of NLRP3–autophagy–SREBP pathways is expected to be a potential therapeutic strategy for DENV infection.

## 2. Materials and Methods

### 2.1. Cell Culture and DENV-2 Virus

HMEC-1 cells, which are an immortalized human microvascular endothelial cell line derived from the foreskin of a male patient (HMEC line 1; Center for Disease Control, Atlanta, GA, USA), were grown at 37 °C under 5% CO_2_ in MCDB131 medium (Gibco/Life Technologies, Carlsbad, CA, USA) and added to 10% fetal bovine serum (FBS), 1 mg/mL hydrocortisone (Sigma Aldrich, St. Louis, MO, USA), 10 ng/mL epidermal growth factor (Gibco Carlsbad, CA, USA), 100 U penicillin, and 100 mg/mL streptomycin (Gibco/Life Technologies, Carlsbad, CA, USA). Cells were harvested with 1000 U/mL trypsin (Sigma Aldrich, St. Louis, MO, USA) and 0.5 mM EDTA (Sigma Aldrich, St. Louis, MO, USA).

Dengue virus serotype 2 new Guinea (DENV-2) stocks were obtained by infecting a C6/36 cell monolayer in 75 cm^2^ tissue culture flasks at 75–85% confluence described by Bustos-Arriaga et al., 2011 [26].

### 2.2. Virus Quantification 

Vero cells were used for DENV-2 titration. These cells were grown in 24-well plates (1 × 10^5^ cells per well) using RMPI medium 1640 (Gibco, Carlsbad, CA, USA) supplemented with 10% FBS, antibiotics, vitamins, amino acids, L-glutamine, and pyruvate (Gibco, Carlsbad, CA, USA), 100 units/mL of each, at 37 °C in a 5% CO_2_ environment. Ten-fold serial dilutions (10^−1^–10^−6^) of cell supernatants in Hanks; salt solution (Gibco, Carlsbad, CA, USA) were used to infect confluent monolayers of Vero cells in 24-well plates. Six tubes were used for serial dilutions. Under sterile conditions, 225 μL of Hanks solution (Gibco, Carlsbad, CA, USA) was added to each tube and 25 μL of the inoculum to the first tube. These were mixed vigorously on a shaker, and so on until 6 dilutions were achieved. The assay was performed in triplicate. Each well was inoculated with 100 μL of the corresponding dilutions. They were incubated for 1 h at 37 °C in a 5% CO_2_ atmosphere and after this, 500 μL/well of Overlay medium (MEM medium with 3% carboxymethyl cellulose (Sigma Aldrich, St. Louis, MO, USA), 2% FBS, and 2 mM L-glutamine (Gibco, Carlsbad, CA, USA) were added. Finally, the cells were incubated at 37 °C for 5 days. After this time, the cells were washed with 1X PBS and fixed with 80% methanol (Sigma Aldrich, St. Louis, MO, USA) with a 10 min incubation at room temperature. Subsequently, the cells were blocked with 1 mL of PBS-5% milk. The virus was identified with a mouse anti pan-Flaviviridae 4G2 antibody (donated by NIH, Rockville, MD, USA) and with an anti-mouse-peroxidase secondary antibody (Thermo Fisher Scientific, Waltham, MA, USA). Finally, True Blue was added (Seractec, Gotingen, Germany), and the cells were incubated for 10 min at room temperature without shaking and the plaques were counted. The calculation was performed as follows: (Log) (Average number of plates × 10^x+1^).

### 2.3. Treatment with Inhibitor Glyburide and Infection Assay

HMEC-1 cells (5 × 10^5^ cells/well) were plated in 6-well plates. After 24 h, the cells were treated with 200 μM Glyburide (Sigma-Aldrich, St. Louis, MO, USA) for 1 h at 37 °C. Subsequently, active or UV-inactivated DENV-2 was added at multiplicity of infection (MOI) of 5 for 2 h. Next, the inoculum was removed, the cells were washed with PBS, fresh growth medium was added, and the cells were incubated at 37 °C for different times for their respective analysis’ requirements.

The rat monoclonal antibody and mouse monoclonal antibody directed against non-structural protein 5 (NS5) and the non-structural 3 (NS3, respectively, previously described by our laboratory [27]. The mouse monoclonal antibodies against SREBP (at dilution of 1:1000) and mouse polyclonal antibody against FAS (at dilution of 1:1000) were obtained from Abcam Limited, Cambridge, UK.

### 2.4. Immunofluorescence Analysis (IF)

HMEC-1 cells were trypsinized and re-suspended in MCDB131 medium (Gibco/Life Technologies, Carlsbad, CA, USA). Briefly, cells were then seeded on glass coverslips (1 × 10^5^ cells/mL). After 24 h, the culture medium was removed, and the monolayer was washed. DENV-2 or DENV-2 UV-inactivated was then added at an MOI of 5. The cells were incubated at 37 °C for 2 h, and the inoculum was then removed. The cells were washed with PBS, and fresh growth medium was added. After time post-infection, HMEC-1 cells were analyzed by immunofluorescence. Briefly, the cells were fixed with 4% paraformaldehyde (Sigma-Aldrich, St. Louis, MO, USA) in PBS for 20 min at room temperature. The cells were then permeabilized with 0.1% Triton-X 100 (Sigma-Aldrich, St. Louis, MO, USA) in PBS and blocked with 10% normal goat serum. The cell monolayer was treated for 1 h with primary mouse monoclonal anti-NS3 (previously described in our laboratory), followed by treatment with fluorochrome-conjugated secondary goat anti-mouse IgG1 (1 mg/mL) (Thermo Fisher Scientific, Waltham, MA, USA).

### 2.5. Immunoblot Analysis

After infection with DENV-2 and treatment with inhibitors, HMEC-1 cells were lysed in RIPA buffer (20 mM Tris-HCl, 150 mM NaCl, 1 mM Na2 EDTA, 1 mM EGTA, 1% sodium deoxycholate, 25 mM phosphate de sodium, 1 mM β-Glycerophosphate) containing the protease inhibitor cocktail for 60 min (Sigma-Aldrich, St. Louis, MO, USA). Total cell lysates were centrifuged at 12,000 rpm for 20 min at 4 °C. Protein concentrations of cell lysates were measured using a protein assay reagent (Bio-Rad Laboratories, Inc., Hercules, CA, USA). For immunoblotting, 30 µg of cell lysate from different conditions were denatured for 10 min at 95 °C. Sample proteins were resolved by SDS-PAGE using different concentrations Tris-HCl gels for 1.5 h at 160 V (Mini-Protean Cell; Amersham Biosciences, Piscataway, NJ, USA) and then electro transferred (120 V for 2 h) onto nitrocellulose membranes (Hybond ECL; GE Healthcare, Little Chalfont, UK). Air-dried membranes were blocked with 5% PBS/Tween-20/milk and then incubated with the appropriate primary antibody overnight, followed by the horseradish peroxidase (HRP)-conjugated secondary antibody in PBS/tween-20 for 1 h. If necessary, the membranes were stripped. After further washing with PBS-Tween 20, the membranes were developed using Super Signal West Pico or West Femto Maximum sensitivity substrate (Thermo Fisher Scientific, Waltham, MA, USA) with a Chemi Doc MP (BioRad Laboratories, Inc., Hercules, CA, USA) imaging capture system. The mouse monoclonal antibody against NLRP3 (AG-20B-0014-C100, 1:1000 dilution) was obtained from AdipoGen Life Sciences, San Diego, CA, USA. The rabbit polyclonal against GAPDH (GTX100118, 1:1000 dilution) was obtained from GeneTex, Inc., Irvine, CA, USA.

The rabbit polyclonal antibody against LC3 (GTX82986) (at dilution of 1:1000) and mouse monoclonal antibody Beclin-1(GTX60413) (at dilution of 1:1000) were obtained from GeneTex, Inc., Irvine, CA, USA. The anti-SQSTM1/p62 antibody (5114) was used at a 1:1000 dilution, obtained from the company Cell Signaling Technology, Danvers, MA, USA. The SREBP antibodies used were the following: SREBP1 (AB3259) dilution 1:1000, SREBP2 (AB30682) dilution 1:1000, and were obtained from Abcam Limited, Cambridge, UK. The NRF2 antibody (dilution 1:1000) is a mouse monoclonal (A-10): sc-365949 from Santa Cruz Biotechnology Inc., Dallas, TX, USA.

### 2.6. Transfection of Reporter Plasmids with GPF-LC3

One day prior to transfection, 1 × 10^5^ HMEC-1 cells were cultured in 24-well plates. Each well was then transfected with 1 μg of pEGFPC1-LC3 in combination with Opti-MEM (Gibco, Carlsbad, CA, USA). Then they were infected with DENV-2 at the indicated times.

### 2.7. MTT Assay

For evaluating glyburide cytotoxicity in HMEC-1 cells, an MTT assay must be performed. This assay measures mitochondrial activity of living cells, due to the reduction of MTT reagent (Thermo Fisher Scientific, Waltham, MA, USA) to formazan blue crystals, produced by mitochondrial metabolism. This reduction indicates cellular metabolic activity. Initially, the HMEC-1 cells were plated in a 96-well plate; 10,000 cells per well. So, we prepared the drug on DMSO [50 mM] (Sigma-Aldrich, St. Louis, MO, USA). Different concentrations between 50 μM and 800 μM were used and incubated for 48 h. After this, the MTT reagent was prepared in RPMI medium without phenol red (0.5 mg/mL). The medium of the cells with the treatment declined and the cells were washed with PBS, followed by 100 ul of the medium with MTT that was previously prepared added by well and incubated for 2 h at 37 °C protected from the light. After, the medium with MTT declined and 100 μL of DMSO was added to each well; finally, the plate was readied in ELISA plate lector to 540 nm of length (Thermo Fisher Scientific, Waltham, MA, USA). The percentage cell viability was estimated by using the following mathematical equation:

$$\%\;\mathrm{cell}\;\mathrm{viability}:\;\frac{\mathrm{average}\;\mathrm{optical}\;\mathrm{densities}\;\mathrm{of}\;\mathrm{tested}\;\mathrm{sample}}{\mathrm{average}\;\mathrm{density}\;\mathrm{of}\;\mathrm{control}}\times 100\%$$

## 3. Results

### 3.1. The Inhibition of NLRP3 Inflammasome Activation by DENV Infection Decreased Viral Replication

Cumulative reports have shown that DENV or some of its proteins such as E or NS1 activate the NLRP3 inflammasome [28,29,30,31]. Previously, our group has described the activation of NLRP3 inflammasome in HMEC-1 cells during DENV-2 infection and this activation is observed also by the expression of a single viral proteins such as NS2A and NS2B both as putative viroporins [28].

To evaluate the implications of inflammasome activation on DENV-2 replication, NLRP3 was inhibited using glyburide, which prevents the activation of NLRP3 [32]. To determine the optimal conditions for glyburide treatment, thus avoiding toxicity in HMEC-1 cells, we decided to test different concentrations close to the reported for other mammalian cells (Supplementary Figure S1).

**Figure 1:**
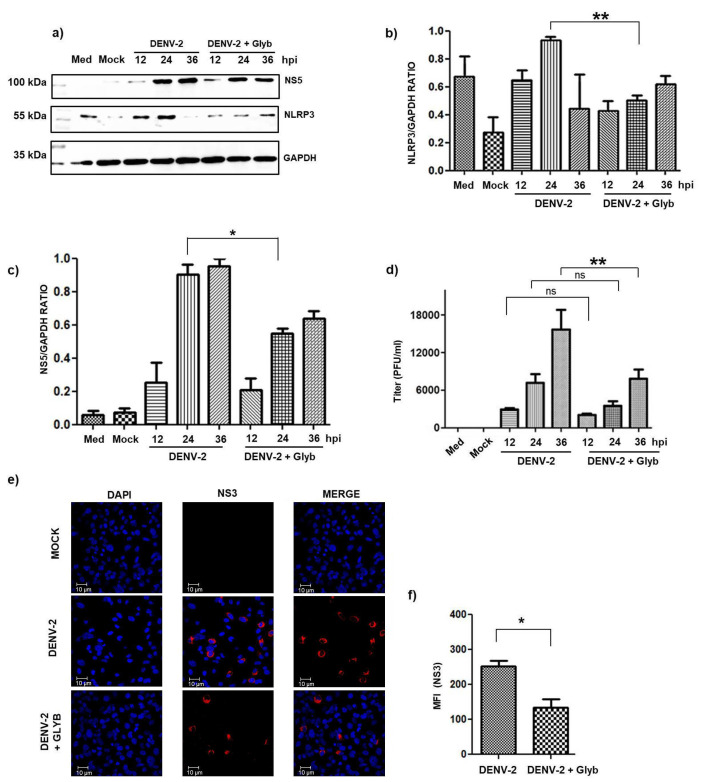
Effects of glyburide on NLRP3, NS5, NS3 viral protein expression, and viral replication. (**a**,**b**) HMEC-1 cells were plated into 24-wells plate, treated with 200 μM of glyburide for 1 h at 37 °C. After treatment, HMEC-1 cells were infected with DENV-2 at an MOI of 5 for 2 h or mock infected (cells treated with UV-inactivated DENV-2); as a negative control, cells were incubated only with culture medium (Med). At 12, 24, and 36 h, the protein expression of NLRP3 were evaluated by Western blotting using specific antibodies and the infection was evaluated by detecting NS5 or NS3 viral proteins. (**c**) NS5 protein expression levels in the Western blot were calculated relative to the expression level of GAPDH. All bars represent the average (+SEM) of three independent experiments. (**d**) Supernatants from infected cells and with Glyb treatment were titrated to analyze the productive infection. (**e**) Immunofluorescence staining was simultaneously performed at different times post-infection (since NS3 viral protein detection). (**f**) Relative mean fluorescence intensity (MFI) of NS3 (red). The samples infected with DENV-2 + Glyb were compared with samples only infected with DENV-2, and each time of infection with Glyb was compared with its respective time without Glyb. We performed two-way ANOVA with post hoc analysis; ns, no significant, * *p* < 0.05 and ** *p* < 0.001.

Thus, we infected HMEC-1 cells with DENV-2 at an MOI of 5, and then cell lysates were analyzed at different times (12, 24, and 36 h) by Western blotting using specific anti-NLRP3 antibodies to follow the infection with anti NS5. During DENV-2 infection, the NLRP3 expression increased considerably in the late phase, along with NS5 (Figure 1a–c). Additionally, as a downstream product of the NLRP3 inflammasome activation pathway, caspase-1 was evaluated, and accumulation of this protein was observed in the DENV-infected cells 24 and 36 h post-infection [28].

In contrast, when HMEC-1 cells were treated with glyburide, both NLRP3 and NS5 were inhibited. To corroborate this observation, we evaluated the effect of glyburide administration on HMEC-1 cells infected with DENV-2 or mock infected at an MOI of 5 for 36 h. Subsequently, the cells were fixed and stained with anti-NS3. We observed a clear decrease in the expression of viral protein NS3 in cells subjected to glyburide treatment (Figure 1e,f) confirming the results observed by WB. Productive viral replication was also evaluated when NLRP3 was inhibited by glyburide treatment in DENV-infected cells. Suppressive effects on DENV replication were clearly observed and statistically significant suppression of DENV-2 titers were detected in contrast to the untreated and mock-infected controls (Figure 1d).

### 3.2. Dengue Virus Induces Autophagy Activation at Early Time Points in HMEC-1 Cells

**Figure 2:**
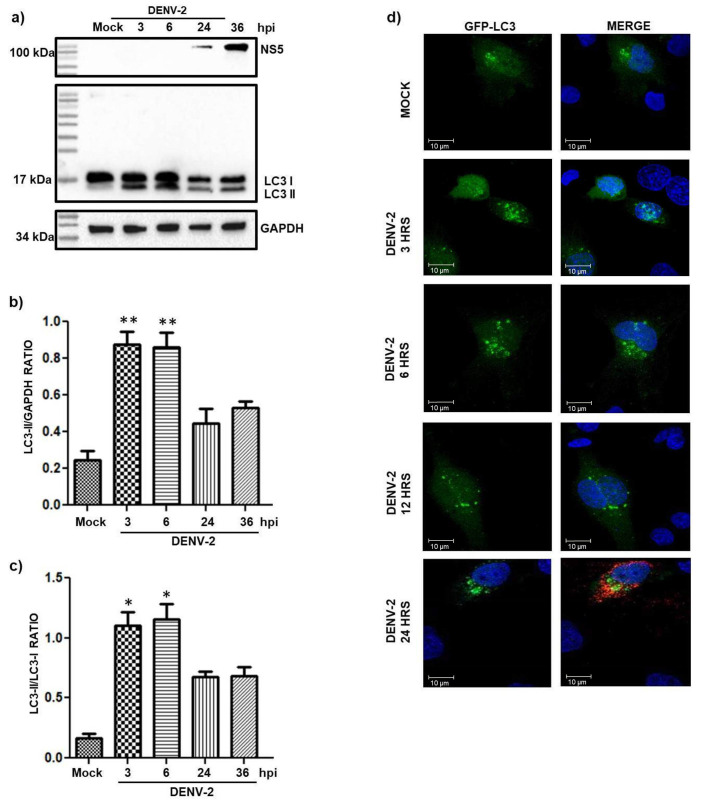
Evaluation of LC3 expression in HMEC-1 cells infected with DENV2 or treated with inactivated DENV-2 analyzed at various time points. HMEC-1 cells were infected with DENV-2 at a multiplicity of infection (MOI) of 5 and analyzed at different time points. NS5 served as an infection control. (**a**) Western blot analysis of NS5, LC3, and GAPDH (loading control) protein levels. (**b**) Quantification of LC3-II normalized with GAPDH. (**c**) Ratio of LC3-II to LC3-I protein levels. Bars represent the average (+SEM) from three independent experiments. Student’s *t*-test was performed to evaluate the statistical significance. * *p* < 0.05 and ** *p* < 0.001. (**d**) HMEC-1 cells were transfected with GFP-LC3 plasmid (green) and subsequently infected or mock infected with DENV-2 at different times. Red fluorescence indicates dsRNA.

Because inflammasome signaling pathways regulate autophagy to maintain a balanced inflammatory response, we aimed to evaluate whether DENV-2 induces autophagy in HMEC-1 cells at different time points (3, 6, 12, 24, and 36 h). Cells were infected with DENV at a multiplicity of infection (MOI) of 5. At each time point, infected cells were harvested, lysed, and analyzed by Western blot to detect LC3 (a well-established autophagy biomarker) using a specific antibody. Our results suggest that DENV-2 triggers the cells not only to form more autophagosomes, as well induce an increase in expression of LC3 at early time points (3, 6, h). Both LC3-I (cytosolic form) and LC3-II (processed form) expression, were observed that increased as well (Figure 2a–c). At late stages (24 and 36 h post-infection), LC3 levels began to decrease.

Furthermore, we performed an immunofluorescence assay on cells transfected with LC3 and subsequently infected with DENV-2 to more clearly demonstrate the presence of fluorescent green LC3 dot structures (representing autophagic vesicles). Analysis of the preparations by confocal microscopy revealed an increase in LC3 dot structures following DENV-2 infection. Infected cells were examined by dsRNA detection. These results confirm the early activation of autophagy (Figure 2d).

### 3.3. Inhibition of the NLRP3 Inflammasome Does Not Reduce Autophagy at Early Time Points

Evidence indicates that some NLRP family members regulate autophagy through its interaction with Beclin-1, a protein involved in autophagy initiation via the NACHT domain [33]. Conversely, many viral infections induce autophagy while inhibiting NLRP3 activation. Regulation of the autophagic process helps to maintain a balance between innate immunity and inflammatory response.

**Figure 3:**
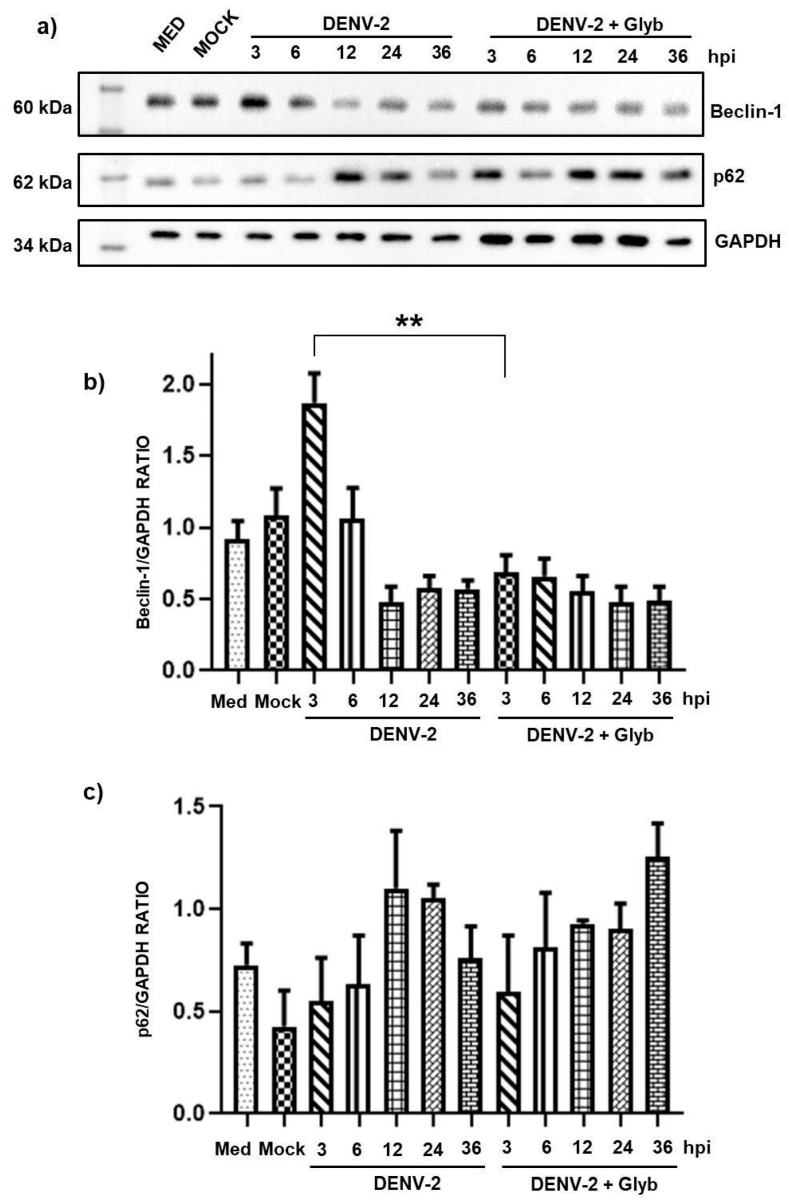
Beclin-1 and p62 expression in DENV-2-infected HMEC-1 cells treated with or without glyburide (200 μM). HMEC-1 cells were infected with DENV-2 at an MOI of 5. (**a**) Beclin-1 and p62 expression at 3, 6, 12, 24, and 36 h post DENV infection and in the presence of glyburide at 3, 6, 12, 24, and 36 hpi. (**b**,**c**) Beclin-1 and p62 protein expression levels in the Western blot were calculated in relation to the expression level of GAPDH. Statistical comparisons were made using two-way ANOVA with post hoc analysis; ** *p* < 0.001.

To investigate whether DENV-induced autophagy is NLRP3-dependent, the HMEC-1 cells were treated with glyburide to inhibit NLRP3 activation. HMEC-1 cells were incubated with either vehicle control or 200 µM glyburide for 21 h. Changes in the expression of Beclin-1 which initiates the formation of the autophagic membrane (phagosome), and p62 (as marker of autophagic flux) were assessed (Figure 3). The same effect was observed for Beclin-1 expression, which increased at early time points (3, 6, and 12 h) and decreased post-infection (24 and 36 h) (Figure 2a), suggesting that DENV regulates Beclin-1 expression during the early stages of infection.

We only observed significant differences in Beclin-1 between 3 h and declining at 12, 24, and 36 h in the absence and presence of glyburide; with respect to the other time points, no significant differences were observed in either Beclin-1 or p62 expression between glyburide-treated and untreated cells, suggesting that early activation of autophagy should be independent of NLRP3 (Figure 3b).

### 3.4. Infection of HMEC-1 Cells with DENV Induces SREBP-1 and SREBP-2 Activation and the Expression of FAS

DENV has been reported to control host lipid metabolism by using cholesterol, triglycerides, and phospholipids at many steps during replication within infected cells [6,34]. Thus, the SREBP-1 and 2 pathways, which are the major components of lipid metabolism, are likely to be affected during DENV infection.

Thus, we evaluated whether DENV-2 infection in HMEC-1 cells triggers the activation of SREBP-1 and 2 molecules. HMEC-1 cells were infected with MOI of 5 and then, at different times, we evaluated the expression of SREBP-1 and 2. Thus, HMEC-1 cells were infected for 12, 24, and 36 h and the course of infection was evaluated by detection of NS5 viral protein expression using immunoblot analysis.

**Figure 4:**
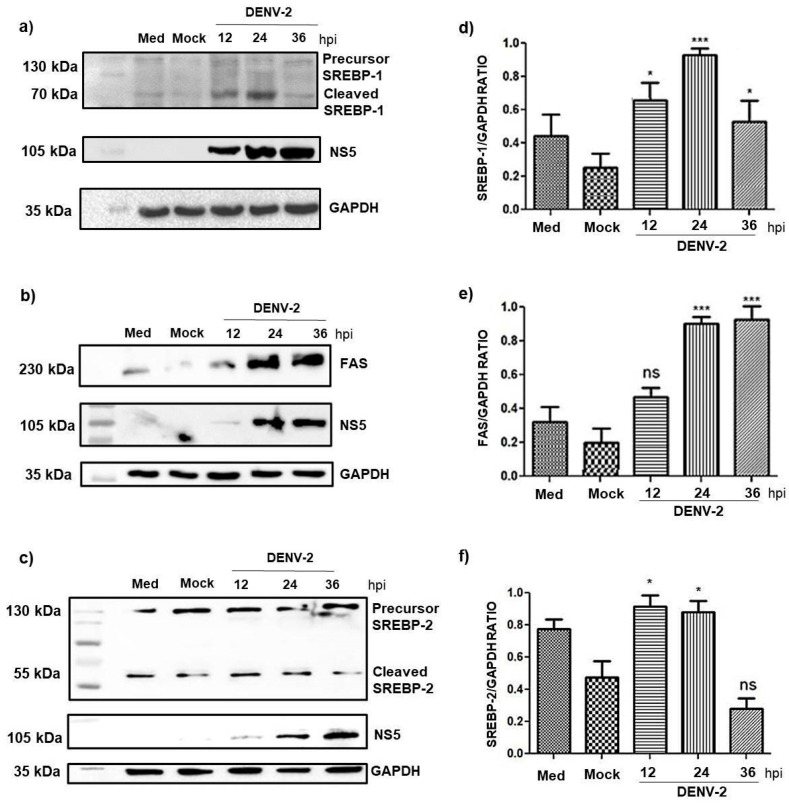
DENV-2 modifies and activates SREBP-1 and SREBP-2. HMEC-1 cells were infected with DENV-2 at an MOI of 5 for 12, 24, and 36 h. (**a**,**c**) Representative Western blot images showing the expression of the precursor and cleaved forms of SREBP-1 and 2, respectively. (**b**) Representative Western blotting images of FAS expression at different time points, along with NS5 as infection control (**d**–**f**) Densitometry analysis of mature SREBP-1, FAS and SREBP-2 in HMEC-1 cells. Bars represent the average (+SEM) of SREBP-1, FAS, and SREBP-2 from three independent experiments. The samples corresponding to 12, 24, and 36 h with DENV-2 were compared with the mock for their respective statistical analysis. Statistical comparisons were made with Student’s test for the determination of the differences between two sample means. ns, no significant, * *p* < 0.05 and *** *p* < 0.001.

Figure 4 shows that during DENV-2 infection, protein expression of SREBP-1 and 2 was induced and levels of molecules proteolytically processed by them also increased. Furthermore, SREBP-1 levels increased following 24 h compared with mock-infected cells or not-treated cells. These data suggest that DENV-2 regulates SREBP-1, which is the transcription factor for lipid synthesis-related genes.

These results suggest that SREBP-1 and 2 play an important role in DENV replication, due to the activation of different molecules involved in lipid metabolism. Considering that mammalian cell lipid profiles are also modified to support DENV replication.

Then we decide to measure the enzyme fatty acid synthase (FAS), which catalyzes the biosynthesis of long-chain fatty acids from acetyl-CoA precursors. The increase in the expression and processing of SREBP-1 protein affected the FAS expression, a major target of SREBP-1. We observed that the expression of active SREBPs, as well as FAS, increased in a time-dependent manner, with higher levels of both detected during the later stages of infection. This result is congruent with that observed in other cellular models and suggests an important role of SREBP-1 and 2 in DENV replication, due to the activation of different molecules involved in lipid metabolism.

### 3.5. NLRP3 Inflammasome Inhibition During DENV-2 Infection Reduces SREBP Activation and Viral Replication

Multiple studies have demonstrated that various viruses stimulate lipogenesis by activating SREBPs, which are present in the cytosol as precursor proteins [15,35].

To investigate the potential link between NLRP3 inhibition and SREBP activation, HMEC-1 cells were treated with glyburide to inhibit DENV-induced NLRP3 activation. This allowed us to assess whether activation of the master SREBP-1 and SREBP-2 is NLRP3-dependent. Thus HMEC-1 cells were incubated with either vehicle control or 200 µM glyburide for 1 h. Levels of both precursor and mature forms of SREBP-1 and SREBP-2 were assessed by immunoblot analysis.

**Figure 5:**
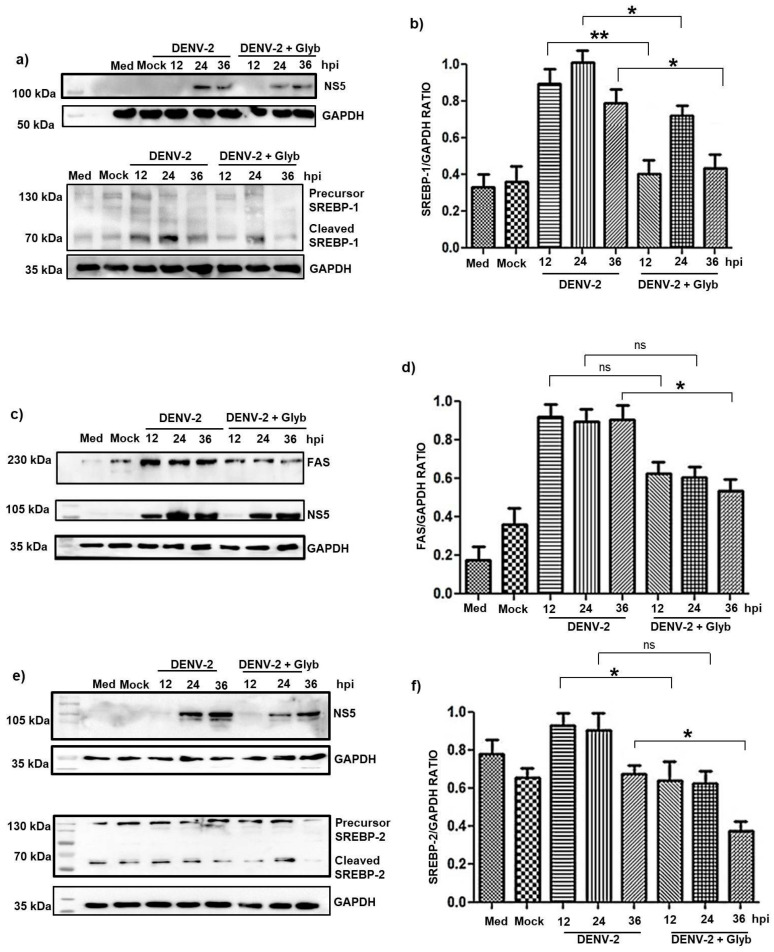
Effects of NLRP3 inflammasome inhibition on SREBP and FAS expression during DENV-2 infection in HMEC-1 cells. HMEC-1 cells were infected with DENV-2 at an MOI of 5 in the presence of glyburide (200 μM). Western blot analysis was performed at 12, 24, and 36 h post-infection). (**a**,**c**,**e**) Representative immunoblots showing SREBP-1, SREBP-2, and FAS protein levels, with NS5 as infection control and GAPDH as a loading control. (**b**,**d**,**f**) Densitometry analysis corresponding to the expression levels of the analyzed proteins. Bars represent the average (+SEM) of SREBP-1, SREBP-2, and FAS from three independent experiments. Samples infected with DENV-2 + glyburide (Glyb) were compared with samples infected without Glyb. Statistical analysis for three independent experiments was performed using two-way ANOVA with post hoc analysis. ns, not significant; * *p* < 0.05 and ** *p* < 0.01.

The results demonstrated that NLRP3 increased both precursor and mature forms of SREBP-1 in a time-dependent manner (Figure 5a,b). In contrast, glyburide treatment inhibited DENV-2-induced NLRP3 activation and reduced SREBP-1 expression and its processed forms (Figure 5a,b). Downregulation of SREBP-1 was associated with decreased DENV-2 replication, as reflected reduction in NS5 protein expression over time (Figure 5a,b).

Similar results were observed for SREBP-2 (Figure 5c,f). Overall, glyburide inhibited SREBP-1 and SREBP-2 by approximately 50% in HMEC-1 cells. Of course, many viruses hijack the lipid metabolism pathways of the host cells to allow viral replication and assembly. It is possible that since the replication decreased with the addition of glyburide, the factor may be degraded.

We next analyzed FAS, a key enzyme in lipid synthesis. As shown in Figure 5b,e, FAS expression increased in a time-dependent manner at 12, 24, and 36 h of infection. Glyburide-mediated NLRP3 inhibition, which reduced FAS expression, consistent with the effects observed on SREBPs (Figure 5b,e).

These data suggest that DENV activates the inflammasome, which can in turn regulate lipid metabolism mediated by SREBP. Furthermore, the data suggest that inflammasome activation culminates in the expression of proinflammatory cytokines and a cellular stress environment. In this sense, IL-1B is a proinflammatory cytokine that activates NFKB, which subsequently results in the expression of lipogenic genes induced by the activation of the master genes SREBP1 and SREBP2.

**Figure 6:**
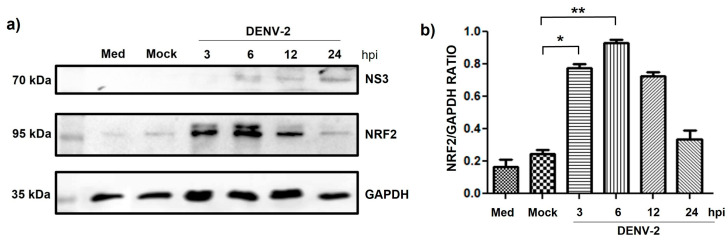
NRF2 expression in HMEC-1 cells in response to DENV-2 infection. (**a**) NRF2 expression in HMEC-1 cells infected with DENV-2 at an MOI of 5 for 3, 6, 12, and 24 h; NS3 was used as an infection control and GAPDH as a loading control. (**b**) Densitometry analysis of NRF2 in HMEC-1 cells. Bars represent the average (+SEM) of nrf2 from three independent experiments. Student’s *t*-test was performed to test the statistical significance. * *p* < 0.05 and ** *p* < 0.001.

We also evaluated NRF2 expression in cells infected with dengue virus to analyze its role in the antioxidant response during infection, in addition to reports that demonstrate its involvement in the activation of autophagy and NLRP3 inflammasome [36,37,38]. Interestingly, the results showed a significant increase in NRF2 expression at early time post-infection, suggesting an early activation of cellular defense pathways against oxidative stress induced by the virus; however, at later times of infection (24 h post-infection), a decrease in NRF2 levels was observed, indicating a possible suppression of this pathway at later stages of infection, which could favor viral replication and redox imbalance in the host cell and the cellular phenomena analyzed (Figure 6).

## 4. Discussion

DENV infection triggers both autophagy and inflammasome activation, processes that are closely linked to the regulation of lipid metabolism and are essential for efficient DENV replication. During its replication cycle, DENV induces autophagy, particularly to modify cellular lipid metabolism. In viral infections, autophagy is rapidly induced as an antiviral response. If the virus evades autophagic degradation, inflammasome activation occurs. These processes are essential for efficient DENV replication. However, the interplay between autophagy dynamics and inflammasome activation during DENV infection has not been systematically investigated.

We have previously demonstrated that viral infection activates the NLRP3 inflammasome in endothelial cells, which is closely associated with increased reactive oxygen species (ROS) as a potential activator [28]. To better understand the interplay between the NLRP3 inflammasome, autophagy, and lipid metabolism during DENV infection, we evaluated the role of inflammasome activation on SREBP induction and autophagy in a HMEC-1 cell model.

DENV infection also activates autophagy, a critical cellular process in viral replication. Autophagy can be triggered by various intra- or extracellular stresses, including nutrient deprivation, endoplasmic reticulum stress, or pathogen infection. In the present study, we examined the expression of LC3, LC3-II (processed form), and Beclin-1, a protein involved in autophagosome initiation. Our analysis revealed elevated levels of these proteins early after infection, followed by a decline at later stages (24 and 36 hpi), indicating DENV-dependent autophagy induction [39,40]. Several factors, including inflammasomes, have been reported to induce autophagy, but the relationship is complex owing to intricate crosstalk. The inflammasome can regulate autophagy through NOD-like receptor (NLR) proteins, which interact with autophagy proteins such as Beclin-1 to modulate autophagy induction [15,41]. The process of autophagy largely depends on the dynamic organization of cellular membranes for its initiation, formation and completion.

DENV modulated the expression of SREBP-1 and SREBP-2 transcription factors, which in turn regulated FAS expression, a key target gene involved in lipid biosynthesis. Once again, these findings strongly suggest that DENV plays a crucial role in modulating lipid metabolism in HMEC-1 cells. Moreover, in agreement with a recent study reporting that SREBP-2 is highly expressed in severe COVID-19 alongside NLRP3 activation [15,41], our data further support the idea that lipid and cholesterol synthesis are crucial processes in the pathology of many viral infections [42].

To investigate the contribution of NLRP3 inflammasomes to autophagy and SREBP protein expression, we inhibited NLRP3 activation with glyburide in DENV-2 infected cells and observed a reduction in the precursor forms of SREBP-1 and SREBP-2, compared with untreated cells. A decrease in FAS expression was also detected in the presence of glyburide. A recent study demonstrated that oxidized low-density lipoprotein (oxLDL) induces NLRP3 activation and is likely responsible for the activating SREBP-1 and its downstream proteins. These observations indicate that inflammatory processes modulate both NLRP3 and SREBP activation [43].

A transient increase in the activation of both SREBP-1 and SREBP-2 has also been reported during hepatitis C virus (HCV) infection, leading to enhanced lipogenesis of cholesterol and membrane lipids [34]. Moreover, a recent study reported that SREBP-2 is highly expressed in severe forms of DENV infection. Several studies have further reported that DENV requires the involvement of distinct lipid molecules to support its viral cycle at different stages of infection [39]. In addition, we previously demonstrated that lipid structures such as lipid rafts play an important role in DENV replication [44].

Furthermore, several studies have reported a link between cellular metabolism and the transcription factor SREBP cleavage-activating protein (SCAP), suggesting that it modulates NLRP3 inflammasome activation [45]. NLRP3, in turn, modulates the innate immune response by promoting the maturation of pro-IL-1β and pro-IL-18 [46]. Consistent with the proposal by Todoric et al. [47], our findings on the effects of DENV suggest the existence of a close crosstalk between cholesterol, lipid synthesis, and inflammatory signaling. The role of NLRP3 inflammasome in lipid synthesis, fatty acid oxidation, and transport across different cells remains to be clarified. However, in the present study, we showed that NLRP3 participates in the regulation of lipogenesis in HMEC-1 cells. Specifically, the inhibition of NLRP3 with glyburide reduced the expression and transcriptional activation of SREBP-1a and SREBP-1c. These findings suggest that NLRP3 functions as a critical regulator of metabolic homeostasis under starvation stress. Nevertheless, the molecular mechanisms directly linking cholesterol metabolic signaling and NLRP3 inflammasome activation remain poorly understood and should be investigated in future research.

As previously reported, there is evidence that SREBP pathway proteins regulate inflammasome activation; however, there is also evidence for inflammasome molecules, such as caspase-1 can promote SREBP activation [48,49]. These findings suggest not merely a crosstalk between the two processes but rather a complex interaction or feedback mechanism.

It has been reported that DENV activates the nuclear factor erythroid-related 2 -like 2 (NRF2) in mononuclear phagocytes through ER stress–PERK (Protein Kinase R-like ER Kinase) pathway at early stages of infection, a process attributed to the viral protease NS2B-NS3. Other viruses, such as HCV, have shown that decreased NRF2 activity induces autophagy via increased ROS production [36]. In the present study, we observed that DENV-2 infection induces autophagy together with ROS-mediated activation of the NLRP3 inflammasome. ROS have been described as crucial for the NRF2-mediated antioxidant response, which counteracts the ROS generated by DENV infection. This mechanism also leads to the overexpression of CLEC5A, enhancing tumor necrosis factor-alpha (TNF-α) production and promoting receptor overactivation [50]. In the present study, we evaluated NRF2 expression in DENV-2–infected cells and found a time-dependent decrease in expression, which could be associated with increased lipid activity, consistent with previous findings. Reports on the role of NRF2 in lipid regulation remain complex [51]. Ferrari et al. reported that the viral protease NS2B-NS3 targets NRF2 for degradation, disrupting the balance between oxidative stress and the NRF2- dependent antioxidant response, which may contribute to inflammation and severity of dengue disease [52]. Thus, degradation of NRF2 could facilitate inflammasome activation, consistent with our results. Moreover, several studies have linked NRF2 deficiency with hepatic lipid accumulation in vivo and in vitro in murine models, as well as with enhanced lipogenesis mediated by increased SREBP-1c activity [53,54]. This observation aligns with our findings of increased SREBP-1c expression and elevated levels of enzymes associated with lipid metabolism. Finally, ROS also interact with autophagy [48]. Autophagy contributes to redox balance by eliminating ROS-damaged molecules and organelles [55]. Notably, multiple proteins have been reported to function as molecular bridges between the Nrf2 pathway and autophagy [56,57].

This study has some limitations. First, we did not perform knockdown experiments for NLRP3 or other components of the inflammasome complex, which would have validated the data generated in this study. Second, glyburide lacks a specific target in the NLRP3 protein. Some reports show some inconsistent effects, since it involves the broader cellular pathway. Lastly, in this study, we did not elucidate the autophagic flux; thus, we did not determine the capacity of the cell to degrade forming autophagosomes.

Overall, our study is the first to suggest that during DENV-2 infection, the activation of SREBP-1 and SREBP-2 is likely dependent on NLRP3 inflammasome activation. This activation favors DENV-2 replication and enhances the expression of FAS and other molecules involved in lipid metabolism.

## 5. Conclusions

We found that NLRP3 plays a critical role in DENV-2 replication, accompanied by the activation of SREBP-1 and SREBP-2 and the expression of genes involved in fatty acid and cholesterol synthesis, such as FAS. Notably, inhibition of DENV-2–induced NLRP3 activation resulted in decreased viral replication, reduced activation of SREBP-1 and SREBP-2, and diminished neutral lipid synthesis.

## Data Availability

The datasets generated during and/or analyzed during the current study are available from the corresponding author on reasonable request.

## Supplementary Materials

The following supporting information can be downloaded at https://www.mdpi.com/article/10.3390/pathogens14121292/s1, Figure S1: MTT assay to evaluate the cytotoxicity of glyburide in HMEC-1 cells. HMEC-1 cells were plating in a 96-well plate. A different concentration between 50 mM and 800 mM were used in the experiment. Glyburide was prepared in DMSO. WO/T: without treatment.

## Abbreviations

The following abbreviations are used in this manuscript:

DENV	dengue virus
NLRP3	NOD-like receptor (NLR) family pyrin domain containing-3
HMEC-1	human microvascular endothelial cells-1
SREBP	sterol regulatory element binding proteins
ER	endoplasmic reticulum
SCAP	SREBP cleavage-activating protein
Nrf2	nuclear factor erythroid-derived 2 -like 2
MOI	multiplicity of infection
hpi	hours post-infection
oxLDL	oxidized low-density lipoprotein
HCV	hepatitis C viral virus
NF-κB	nuclear factor kappa-light-chain-enhancer of activated B cells
ROS	reactive oxygen species
TNF-α	tumor necrosis factor-alpha
ULK1	unc-51 like autophagy activating kinase 1
PI3K	phosphatidylinositol 3-kinase
PIK3C3/Vps34	phosphatidylinositol 3-kinase catalytic subunit type 3/vacuolar protein-sorting 34
ATG5	autophagy-related gene 5
FAS	fatty acid synthase
RVA	rotavirus A
HMGCR	3-hydroxy-3-methylglutaryl-CoA reductase
