# Supplementary figures and images for: Interplay Between NLRP3 Activation by DENV-2 and Autophagy and Its Impact on Lipid Metabolism in HMEC-1 Cells
Source: Pathogens. 2025 Dec 16;14(12):1292. doi: 10.3390/pathogens14121292 (PMC12735470; doi:10.3390/pathogens14121292)

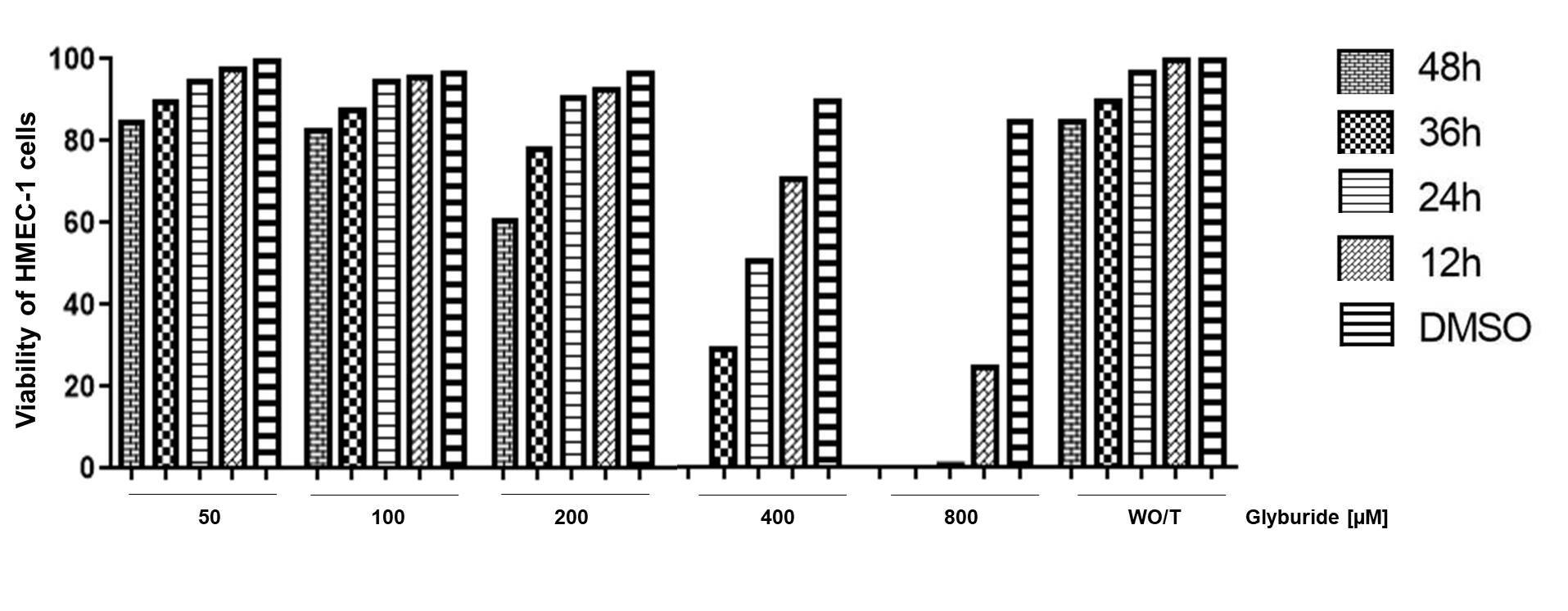

Supplement: Supplementary file 1 [file pathogens-14-01292-s001.zip › pathogens-3934902-supplementary.jpg]
